# Assessing Biotic and Abiotic Interactions of Microorganisms in Amazonia through Co-Occurrence Networks and DNA Metabarcoding

**DOI:** 10.1007/s00248-021-01719-6

**Published:** 2021-02-18

**Authors:** Camila Duarte Ritter, Dominik Forster, Josue A. R. Azevedo, Alexandre Antonelli, R. Henrik Nilsson, Martha E. Trujillo, Micah Dunthorn

**Affiliations:** 1 Eukaryotic Microbiology, University of Duisburg-Essen, Universitätsstrasse 5 S05 R04 H83, D-45141 Essen, Germany; 2 Department of Ecology, University of Kaiserslautern, D-67663 Kaiserslautern, Germany; 3 Programa de Coleções Científicas Biológicas, Coordenação de Biodiversidade, Instituto Nacional de Pesquisas da Amazônia, Manaus, 69060-000 Brazil; 4 Gothenburg Global Biodiversity Centre, Box 461, SE-405 30 Göteborg, Sweden; 5 Department of Biological and Environmental Sciences, University of Gothenburg, Box 463, SE-405 30 Göteborg, Sweden; 6 Royal Botanic Gardens, Kew, TW9 3AE, Richmond, Surrey, UK; 7 Department of Plant Sciences, University of Oxford, South Parks Road, Oxford, OX1 3RB UK; 8 Departamento de Microbiología y Genética, Campus Miguel de Unamuno, 37007 Salamanca, Spain; 9 Centre for Water and Environmental Research (ZWU), University of Duisburg-Essen, Essen, Germany

**Keywords:** Bacteria, Biodiversity, Fungi, Metabarcoding, Protists, Tropics

## Abstract

Species may co-occur due to responses to similar environmental conditions, biological associations, or simply because of coincident geographical distributions. Disentangling patterns of co-occurrence and potential biotic and abiotic interactions is crucial to understand ecosystem function. Here, we used DNA metabarcoding data from litter and mineral soils collected from a longitudinal transect in Amazonia to explore patterns of co-occurrence. We compared data from different Amazonian habitat types, each with a characteristic biota and environmental conditions. These included non-flooded rainforests (terra-firme), forests seasonally flooded by fertile white waters (várzeas) or by unfertile black waters (igapós), and open areas associated with white sand soil (campinas). We ran co-occurrence network analyses based on null models and Spearman correlation for all samples and for each habitat separately. We found that one third of all operational taxonomic units (OTUs) were bacteria and two thirds were eukaryotes. The resulting networks were nevertheless mostly composed of bacteria, with fewer fungi, protists, and metazoans. Considering the functional traits of the OTUs, there is a combination of metabolism modes including respiration and fermentation for bacteria, and a high frequency of saprotrophic fungi (those that feed on dead organic matter), indicating a high turnover of organic material. The organic carbon and base saturation indices were important in the co-occurrences in Amazonian networks, whereas several other soil properties were important for the co-exclusion. Different habitats had similar network properties with some variation in terms of modularity, probably associated with flooding pulse. We show that Amazonian microorganism communities form highly interconnected co-occurrence and co-exclusion networks, which highlights the importance of complex biotic and abiotic interactions in explaining the outstanding biodiversity of the region.

## Introduction

Patterns of co-occurrence among species have traditionally been explained by the species’ similar environmental preferences or tolerances, such as pH range, temperature, habitat type, and similar biogeographic histories generating coincident distributions [1–3]. Species co-occurrence can also be related to biotic interactions, such as predation and parasitism [4]. Such patterns can be described with the use of ecological network analyses, an important tool to investigate community structure and the function and stability of ecosystems [5]. Identifying co-occurrence patterns is essential to grasp the potential ecological interactions and their impact on biodiversity and ecosystem functioning [6, 7].

In the tropics, patterns of co-occurrence can be extremely complex due to the high diversity of species inhabiting different kinds of habitats. The study of species interactions in tropical environments has been largely neglected (but see [8–12]). Most studies of co-occurrences have focused on groups of symbiotic organisms, such as trees and mycorrhizas [13–15] and trees and seed dispersers [16, 17], and are largely centered around mutualism [18] and parasitism [19–21]. However, many other interactions among organisms may play a crucial role in biotic and/or abiotic interactions in the tropics, such as those related to nutrient cycling and organic matter decomposition. Unveiling these potential interactions is important to shed light on the ecological dynamics and ecosystem functions of biodiverse regions.

Microorganisms account for the majority of biodiversity in any environment, including the tropics [22], being crucial for ecosystem dynamics such as nutrient recycling [23, 24] and much of organic soil decomposition [25, 26]. A negative correlation between Amazonian soil organic carbon and soil biodiversity has been found, which could be explained by high soil biodiversity accelerating organic decomposition and subsequently locking carbon in aboveground biomass [27].

Amazonia is the world’s largest tropical forest, covering ca. 3.6% of the global surface while comprising approximately 40% of all rainforest. It harbors 10% of the world’s known biodiversity [28] and potentially a quarter of the world’s terrestrial species [29]. Amazonia is home to a large number of coexisting and potentially interacting species, which probably make up one of the most complex webs of life on Earth. As such, Amazonia provides a wide range of ecosystem services through its high above- and belowground biodiversity [30], including water cycling and carbon storage [31–33], which are mediated through biotic and abiotic interactions. Amazonia can be divided into four main phytophysiognomies (hereafter habitats): non-flooded rainforests (terra-firme), forests that are seasonally flooded either by fertile white waters (várzeas) or by unfertile black waters (igapós), and naturally open areas associated with white sand soil (campinas). The geographical location, the degree of connectivity and isolation, and the soil characteristics of those four main habitat types are important determinants of their biota [34–36], which may consequently determine species co-occurrence patterns.

Recent studies have shown that Amazonian habitat types influence the composition of microbial communities [36]. Microorganism diversity is the richest in campina habitats [36, 37], in contrast to known patterns of macro-organisms that show low diversity in these habitats [38, 39]. Campinas have a highly specialized biota, probably explained by multiple stressors that affect the habitat, such as poor soils and insular distribution [38]. Likewise, the flooded habitats have a highly specialized biota due to stress induced by seasonal flooding [40]. Furthermore, the soil characteristics of all these habitats are different and explain in part the composition of the biota, with pH and organic carbon being the most important soil properties for microorganism richness and turnover [27]. Most microorganisms seem to occur in just one locality and habitat type [36], indicating a potentially high degree of local specialization or low detectability. The diversity of microbial communities and their composition in Amazonia have already been investigated thanks to advances in high-throughput sequencing (HTS) methods [27, 36, 41–45]. However, most of these studies focused on single properties of the communities, such as alpha and beta diversity, and less on the potential interactions between microorganisms as revealed through ecological networks. The integration of graph theory and network analysis into metabarcoding studies allows the exploration of complex community interactions beyond descriptive diversity patterns [46]. Furthermore, graph properties such as density (the rate of connection between nodes), transitivity (probability for the network to have adjacent nodes interconnected), and modularity (the degree of segregation between different modules) from networks may allow an assessment of an ecosystem’s integrity and resilience [47–50].

**Fig. 1:**
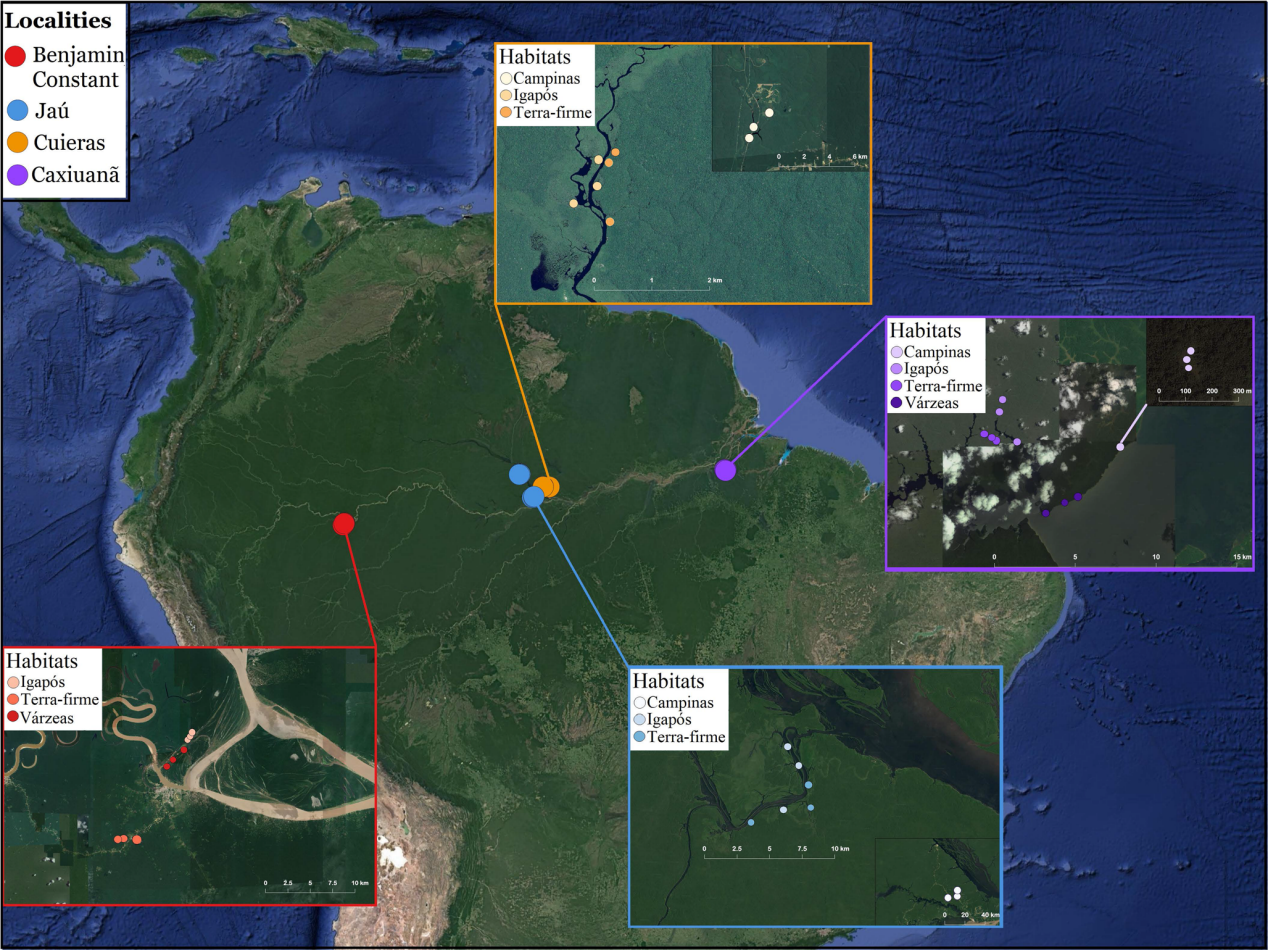
Map of sampling localities. Inset panels show a zoom-in for each locality with the plot distributions. Localities are shown in different colors, while habitat types have different color tones. The sampling covers all major habitat types in Amazonia and spans over 2000 km from west to east

Here, we explore the potential interactions of microorganisms (bacteria, fungi, and protists) and some plants and metazoans in soil using co-occurrence network analysis in four localities in Amazonia. These localities are separated by wide rivers and long distances (Fig. 1) and have previously been examined for diversity using both richness and the effective number of operational taxonomic units (OTUs [51]) [27, 36]. We also test for the importance of environmental filters based on the soil physicochemical properties quantified in each sampled plot [27]. Based on previously identified patterns of OTU richness and distribution in each habitat [36], we expect that the co-occurrence networks will show different structures depending on habitat type. We formulated the following specific hypotheses, based on the current literature: H_1_: The Amazonia-wide network will be mostly composed of organisms associated with organic decomposition; H_2_: Environmental soil properties, especially pH and organic carbon [27, 52], will be the most important factors, acting as key nodes, to explain co-occurrence in all the networks; and H_3_: The habitat-specific networks will show different network structures, with the presence of flood pulse (the periodic inundation of floodplains along certain rivers) as the main factor explaining network properties. We expect that the high environmental stress in seasonal flooding forests will act as an abiotic filter, keeping the same set of specialized organisms that co-occur for long periods of time, thus, resulting in a dense and highly connected network (high transitivity, low modularity).

## Material and Methods

### Sampling and Sequencing

We sampled four main localities along a longitudinal transect (Fig. 1) in November 2015, a month of the dry season without inundation of seasonally flooded forests (around 5 months after inundation peak). The data was previous published in Ritter et al. [27, 36, 37]. In each locality, we sampled all of the four habitats (terra-firme, campinas, várzea, and igapó) whenever they were present (Fig. 1). We used data from soil and litter metabarcoding of the V3–V4 region of the ribosomal small subunit 16S rRNA, targeting bacteria with primers described in Klindworth et al. [53]; the V7 region of the ribosomal small subunit 18S rRNA with primers described in Guardiola et al. [54], as well as the mitochondrial cytochrome c oxidase subunit I (COI) gene, using the forward primer described in Leray et al. [55] and the reverse primer described in Meyer [56], both targeting eukaryotes. Details of sampling and sequencing can be found in Ritter et al. [36, 37]. Briefly, we filtered the low-quality sequences, discarded the chimeras, and clustered sequences into OTUs at 97% similarity using a “greedy” algorithm that performs chimera filtering and OTU clustering simultaneously with the USEARCH / UPARSE v. 9.0.2132 Illumina paired reads pipeline [57].

### Classification of OTUs

We classified OTUs into two categories by habitat occurrence: “specialist” if they occurred in only one habitat (e.g., only in campinas) and as “generalist” if they occurred in at least three habitats. These definitions follow earlier studies on the distribution of organisms in Amazonia as well as previous analyses of our data [36]. To classify the OTUs and produce the figures, we used the tidyverse v.1.2.1 [58] and ggplot2 v.3.2.1 [59] R packages in R v.3.6.0 [60].

We assigned OTUs according to the taxonomic classification published in Ritter et al. [36]. As far as possible, we used the class or order for the classification of the OTU. We assessed the bacterial OTUs for the functional traits using the FAPROTAX database [61], which we applied to all bacteria in our networks. Since several bacteria were assigned to multiple functional traits, we reviewed all classifications and assigned them to the most probable or the dominant functional trait based on the literature and our professional experience. For protists, we used the study of Adl et al. [62] as a reference. For fungi, we used the functional classification published for the same data as in Ritter et al. [63].

### Co-occurrence Network Analysis

Network analyses were performed using abundance matrices as recommended [3] and the null model strategy described in Connor et al. [64], which is regarded as the most reliable strategy for inferring both co-occurrence and co-exclusion relationships in HTS datasets of microbial organisms [65]. The analysis was implemented using the NetworkNullHPC script (https://github.com/lentendu/NetworkNullHPC). Since our study included data from three independent sequencing runs, a normalization of the OTU-to-sample matrices was done to ensure comparability between the data. Drawing conclusions from data of independent sequencing runs without normalization is prone to misinterpretations because of technical biases, such as excessive false-positive correlations among sequencing data of the same runs. To account for this problem, NetworkNullHPC provides several options for normalizing data from which we chose the default one. This option considers the expected sequencing depth of the datasets (by default set to half of the median read abundance determined through all samples) and then performs a normalization by scaling the sum of the read counts in each sample to the expected sequencing depth. After normalization, NetworkNullHPC conducted the null model strategy by Connor et al. [64], which consists of (i) a first class of null models, where noise is added to every entry of the OTU matrix; (ii) the noise-added matrix is permuted, and the distribution of similarity scores in the permuted matrix is used to set the lower bound for the threshold; (iii) the threshold is applied to derive the observed network. This network is used to construct the second class of null models, Erdős-Rényi, based on the average degree, and the Chung-Lu model, based on the average degree distribution of the network. This method calculates a consensus network, which contains every pairwise interaction that is present in at least 90% of the Monte Carlo samples to define the Spearman correlation threshold [64]. Furthermore, only OTUs whose read abundances were higher than 10% of the median read abundance determined through all samples were kept for the network analyses. Low-abundant OTUs (including singleton OTUs with a read abundance of (1) were discarded at this point. In the network analyses, OTUs are represented as nodes, and a statistically significant Spearman rank correlation, calculated through the null models described above, between two OTUs is represented by an edge between the respective OTUs. The networks contain only OTUs that have a significant co-occurrence or co-exclusion with at least one other OTU. We further included soil physicochemical properties (soil texture, exchangeable bases, pH, organic carbon, phosphorus, and aluminum) of each sample from Ritter et al. [27] as environmental parameters into the network analysis for considering significant correlations between OTUs and abiotic variables.

We first produced a network with all OTUs recorded in all samples. We then compared the patterns of co-occurrence in each habitat type and produced networks for each habitat separately using all OTUs recorded in a determined habitat. Due to possible regional variation and differences in the number of replicates among habitats, we also constructed networks for each habitat within each locality. Finally, to obtain a better understanding of significant co-occurrences within different taxonomic groups, we calculated networks using OTUs of all samples for bacteria, all eukaryotes, fungi, protists, and metazoans separately. We then used the tidyverse and igraph v.1.2.4.1 [66] R packages to combine the resulting classification matrices to visualize and explore the networks with the interactive platform Gephi v.0.9.2, using the Yifan Hu layout [67].

For comparing co-occurrence patterns within microorganism communities of the different habitats, we analyzed properties of the network structure by applying several measures of the graph theory [68] (for a schematic representation of some network properties, see Fig. S1): (1) the *maximum component* of the network, which is the subgraph that comprises the largest number of nodes (OTUs); (2) the *diameter*, which is the number of edges connected in the maximal shortest path length of the network and describes the size of the maximum component; (3) the *average path length* of the network, where length values closer to 1 indicate a more direct association of OTUs with each other; (4) the *transitivity*, which is the probability of two random nodes (OTUs) being directly or indirectly connected. This measure provides an indication of the clustering in the whole network, representing the presence of tripartite relationships (nodes connected by more than one path); high transitivity means a tightly connected community and might be indicative of degradation pathways or niche filtering [69]; (5) two *modularity* scores that assess the community structure in the network and show if the community consists of smaller groups of highly associated OTUs that are poorly associated with other OTUs. Modules may indicate different niches and have been used to study habitat preferences [69]. For this metric, we first calculated the modularity of the network and in a second step also identified Louvain communities in the networks, which are groups of highly associated OTUs. Inside these groups, we calculated the modularity (LCmodularity) [70].

In addition to those measures, we calculated the *assortativity*, which is the tendency of nodes that share a specific attribute to be connected within the network, for each main taxonomic group [71]. In other words, we inferred the chance of OTUs of each taxonomic group being more connected among themselves than with OTUs of other taxonomic groups. Furthermore, we also calculated the degree of each node, that is, the number of direct connections it has to other nodes (Fig. S2 and S3). Finally, we calculated the *key nodes*, which are articulation point nodes that have a high betweenness centrality (the extent to which a node lies on paths between other nodes), and identified the OTUs that maintain the network structure and, potentially, mediate several of the processes in the community. Key nodes may represent keystone species, and their removal can cause the ecosystem to collapse [69]. These structural properties of the network allow comparison among complex networks, making it possible to compare the networks among habitats and therefore test our hypotheses. All network analyses were calculated using the igraph R package.

## Results

**Fig. 2:**
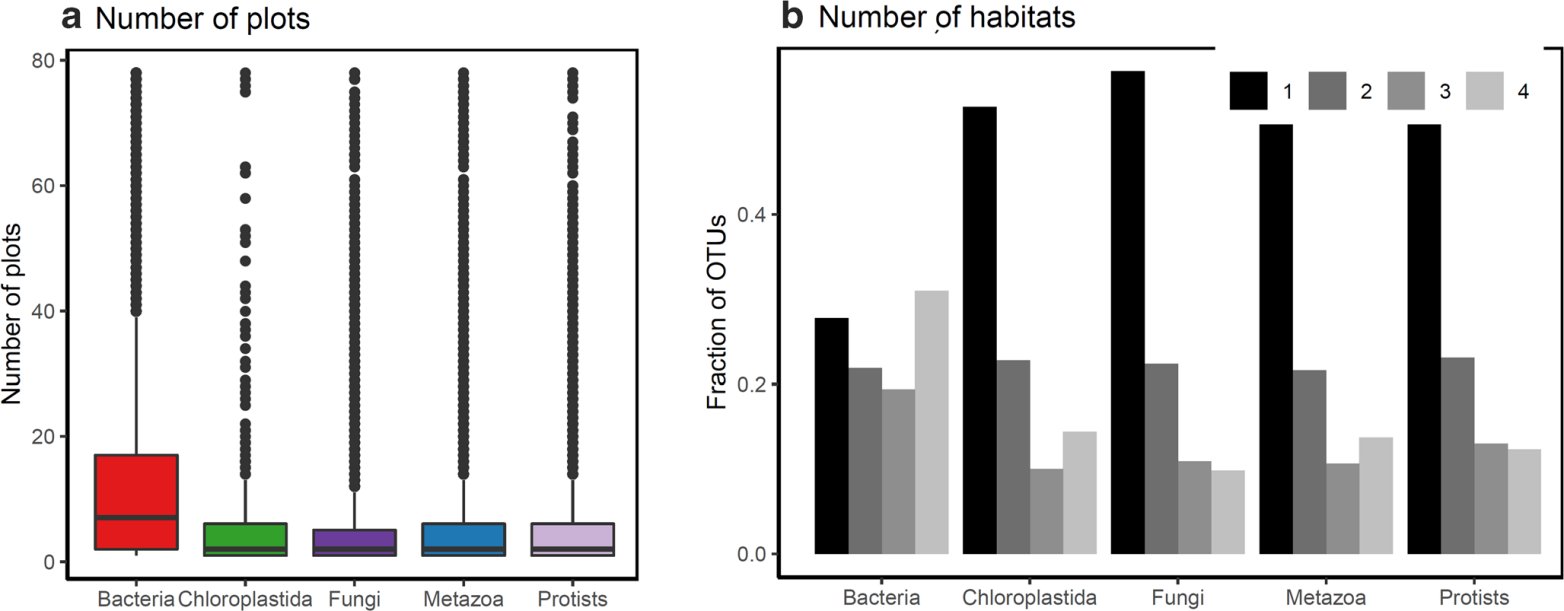
The proportion of OTUs per sampling plot. **a** Boxplot for the main taxonomic groups showing the mean and 95% quartiles of the occurrences of OTUs per sampling plot. **b** The total number of main taxonomic OTU groups per number of habitats. Most OTUs occur in fewer than five plots. Bacteria are more generalist (occurring in ≥3 habitats) than eukaryotic groups

We obtained a total of 39,351 OTUs, of which 9943 (25%) were classified as bacteria, 6750 (17%) as fungi, 5568 (14%) as protists, 5107 (13%) as metazoans, and 438 (1%) as chloroplastida. Many OTUs (10,443; 26%) could not be classified to any resolved taxonomic level. We decided to keep them because even though some of them could be technically compromised, after all clean steps we consider the vast majority should be true OTUs not yet represented in available databases, considering the limited reference sequences for Amazonia. The bacteria were composed mostly of generalist OTUs (~50%), in contrast to a lower proportion of generalists among eukaryotes (~25%; Table 1; Fig. 2).

**Table 1:** Number and proportion (in brackets) of OTUs and their classification by habitat (generalist ≥3 and specialist = 1). More bacterial OTUs were classified as generalists (~50%) than was the case for eukaryotes (~25%), while eukaryotes OTUs were more specialist (~50%) than prokaryotes (~25%)

Group	Total	Generalist	Specialist
All	39,351	10,875 (28%)	20,495 (52%)
Bacteria	9943	5007 (50%)	2761 (28%)
Protists	6568	1659 (25%)	3395 (52%)
Fungi	6750	1394 (21%)	3843 (57%)
Metazoa	5107	1242 (24%)	2763 (54%)
Chloroplastida	438	107 (24%)	231 (53%)
Unknown	10,443	1422 (14%)	7477 (72%)

The network analysis considering all samples resulted in 72,577 edges (statistically significant correlations between pairs of OTUs) involving 2350 OTUs (6% of the total), of which 61,145 edges involved 2340 OTUs for the co-occurrence network, and 11,432 edges involved 1343 OTUs for the co-exclusion network. The Spearman’s rank correlation threshold, calculated based on the null models, was set to 0.43 for the co-occurrence and − 0.42 for the co-exclusion networks. The number of OTUs, nodes, edges, and the Spearman’s rank correlation threshold for habitat-specific networks are shown in Table S1.

Overall, our Amazonian network comprised highly connected OTUs. The maximum component comprised 2337 OTUs (co-occurrence = 2327, co-exclusion = 1339) with a diameter (the maximal shortest path) of eight edges (both co-occurrence and co-exclusion). The transitivity—the measure of the clustering in the network—was 0.386 (co-occurrence = 0.432, co-exclusion = 0). The modularity score (range of −1 to 1) was low, −0.000002 (co-occurrence = −0.000008, co-exclusion = −0.00003), and the LC modularity was 0.469 (co-occurrence = 0.574, co-exclusion = 0.444).

The assortativity of the network showed that bacterial OTUs are mainly connected with other bacterial OTUs; similarly, eukaryotic OTUs are mainly connected with each other (Table S2). When we divided the bacteria by phyla and eukaryotes into fungi and protist realms, and into the metazoan kingdom, this tendency was still observed with few exceptions, that is, OTUs of one phylum or group will be more connected with OTUs of the same phyla (Table S2). Due to the limited number of OTUs classified as Chloroplastida, we did not calculate assortativity for this group.

For the degree attribute, the co-occurrence network showed that several bacterial groups were highly connected and served as hubs (Fig. S2A). The highest degree in the co-exclusion network, that is the nodes that were most connected (with more edges) with other nodes, was observed for several soil properties (pH, sodium [Na], potassium [K], phosphorus (P), calcium [Ca], magnesium [Mg], the sum of all exchangeable bases [SB], exchangeable aluminum [Al and H + Al], aluminum saturation index [m], Base Saturation Index [V], the effective cation exchange capacity [t], the cation exchange capacity [T], and soil texture), and OTUs classified as Acidobacteria, Actinobacteria, and Proteobacteria (Fig. S2B). On top of having a high degree, several soil properties (base saturation index, cation exchange capacity, exchangeable bases, exchangeable aluminum, aluminum saturation index, pH, and soil texture) were also identified as key nodes (Table S3) in the co-exclusion network (Fig. S2B). The values of each score by habitat are provided in Table 2. We found that a total of 124 OTUs were classified as key nodes, mostly bacteria (61%, Table S3). For networks for each taxonomic group, see Figs. S4–S7.

**Table 2:** Properties of habitat-specific networks for all samples in the same habitat (all) and for habitat within each locality (in west-to-east order: *BC* Benjamin Constant, *JAU* Jaú, *CUI* Cuieras, and *CXN* Caxiuanã)

		Campinas				Terra-firme					Várzea			Igapó				
		All	JAU	CUI	CXN	all	BC	JAU	CUI	CXN	all	BC	CXN	all	BC	JAU	CUI	CXN
Max component	**All**	728	16	14	38	972	14	14	23	38	48	13	16	820	13	11	8	10
	**Co-occur**	666	16	12	34	920	11	11	19	34	16	12	13	745	12	9	7	10
	**Co-excl**	147	16	14	38	322	14	14	23	38	31	13	16	327	13	11	8	10
Diameter	**All**	17	1	1	1	14	1	1	1	1	11	1	1	13	1	1	1	1
	**Co-occur**	20	1	1	1	24	1	1	1	1	6	1	1	17	1	1	1	1
	**Co-excl**	16	2	2	2	15	2	2	2	2	13	2	2	15	2	2	2	2
Average path length	**All**	5.16	1	1	1	4.38	1	1	1	1	4.24	1	1	4.4	1	1	1	1
	**Co-occur**	6.95	1	1	1	5.85	1	1	1	1	1.95	1	1	5.72	1	1	1	1
	**Co-excl**	6.05	1.55	1.6	1.68	5.24	1.56	1.53	1.58	1.68	4.04	1.47	1.55	5.29	1.61	1.52	1.4	1.5
Transitivity	**All**	0.44	1	1	1	0.4	1	1	1	1	0.41	1	1	0.39	1	0.99	1	1
	**Co-occur**	0.46	1	1	1	0.44	1	1	1	1	0.5	1	1	0.42	1	0.98	1	1
	**Co-excl**	0	0	0	0	0	0	0	0	0	0	0	0	0	0	0	0	0
Modularity	**All**	0.04	0.96	0.96	0.43	0.01	0.96	0.49	0.45	0.43	0.02	0	0.04	0.002	0	0.37	0.08	0.04
	**Co-occur**	0.06	0.95	0.96	0.47	0.02	0.96	0.96	0.51	0.47	0.06	0.05	0.06	0.004	0	0.62	0.14	0.04
	**Co-excl**	0.26	0.96	0.96	0.89	0.087	0.96	0.15	0.95	0.89	0.16	0.05	0.07	0.003	0.02	0.05	0.96	0.23
LC modularity	**All**	0.64	0.96	0.96	0.86	0.63	0.96	0.95	0.94	0.86	0.89	0.95	0.93	0.56	0.96	0.97	0.97	0.97
	**Co-occur**	0.65	0.95	0.96	0.84	0.7	0.96	0.96	0.94	0.84	0.92	0.96	0.94	0.6	0.96	0.97	0.97	0.96
	**Co-excl**	0.78	0.96	0.96	0.9	0.63	0.96	0.89	0.95	0.9	0.88	0.95	0.91	0.66	0.95	0.96	0.96	0.96
Key nodes	**All**	65	0	0	0	90	0	0	0	0	34	0	0	77	0	0	0	0
	**Co-occur**	28	0	0	0	73	0	0	0	0	22	0	0	59	0	1	0	0
	**Co-excl**	28	14	15	9	73	12	5	15	9	22	7	8	59	13	18	23	7

**Fig. 3:**
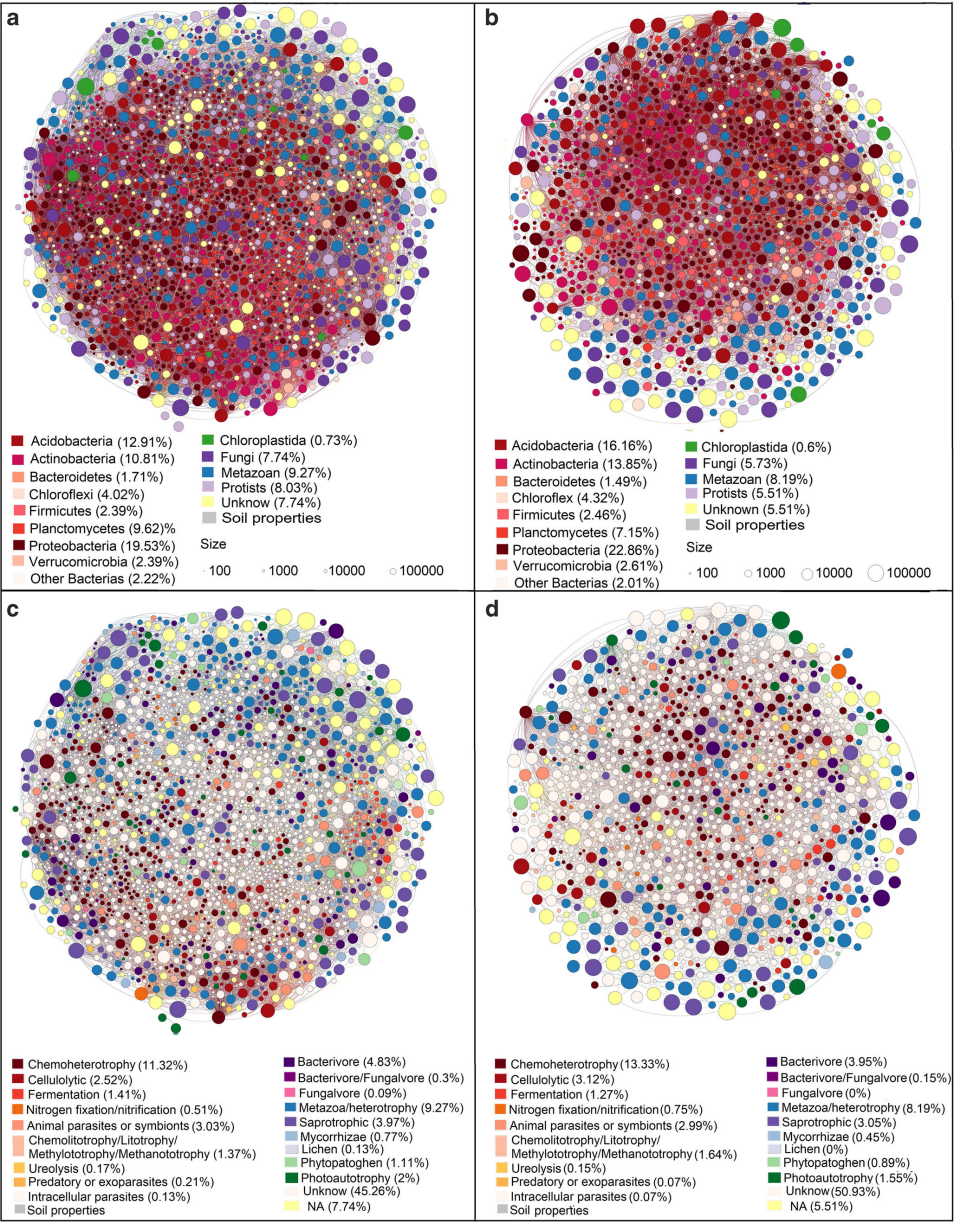
Classification networks for Amazonian organisms, depicting taxonomic classification for **a** co-occurrence, **b** co-exclusion, and functional traits for **c** co-occurrence and **d** co-exclusion. Each OTU is represented by a node (circle) colored according to its taxonomic (**a** and **b**) or functional traits (**c** and **d**). The lines represent the edges connecting the OTUs. The size of the node represents the OTU abundance. The co-occurrence network is dominated by bacteria; however, fungi and metazoans were more abundant. Most of the functionally classified OTUs are associated with organic decomposition

Although bacteria accounted for approximately half of the number of OTUs compared to eukaryotes in our data, they dominated the networks, stressing the importance of prokaryotes in soil communities. The networks are composed mostly of Proteobacteria (Alphaproteobacteria) and Acidobacteria (Fig. 3a). Other groups such as Chloroplastida, fungi, protists, and metazoans are also represented. Most of the OTUs classified as Chloroplastida belong to Magnoliophyta, mainly from the orders Fabales, Liliopsida, and Malpighiales. Most fungi are Dikarya, the majority belongs to Ascomycota and the remainder to Basidiomycota (Fig. S5). The metazoans are mostly comprised of Hexapoda (Fig. S6). Most protists belong to the supergroup SAR (mostly Rhizaria, followed by Alveolata, and Stramenopiles) followed by Amebozoa (Fig. S7). The co-exclusion network is smaller but still features all taxonomic groups (Fig. 3b). For networks scaled to indicate the degree of each node, see Fig. S2.

Our first hypothesis (H_1_)—that most OTUs in the network will be associated with organic decomposition—was supported. Most bacteria were classified as chemoheterotrophic. For bacterial metabolism, there is a combination of respiration and fermentation, which are complementary in the context of organic decomposition. Also, most bacteria were classified as acidophilic or acid tolerant (Fig. 3 and S8). Most fungi were saprotrophic, and most protists were bacterivores (Fig. 3).

Our second hypothesis (H_2_)—that some soil properties (mainly pH and organic carbon) will be important factors in explaining the co-occurrence networks—was partially supported. Soil properties were important for the co-exclusion network and less important for the co-occurrence network (Fig. S2, Table S3). For the network considering all OTUs, only organic carbon (C) and base saturation index (v) were key nodes in explaining co-occurrence, whereas several other soil properties were key nodes for the co-exclusion, notably soil texture, exchangeable bases, aluminum, and pH.

**Fig. 4:**
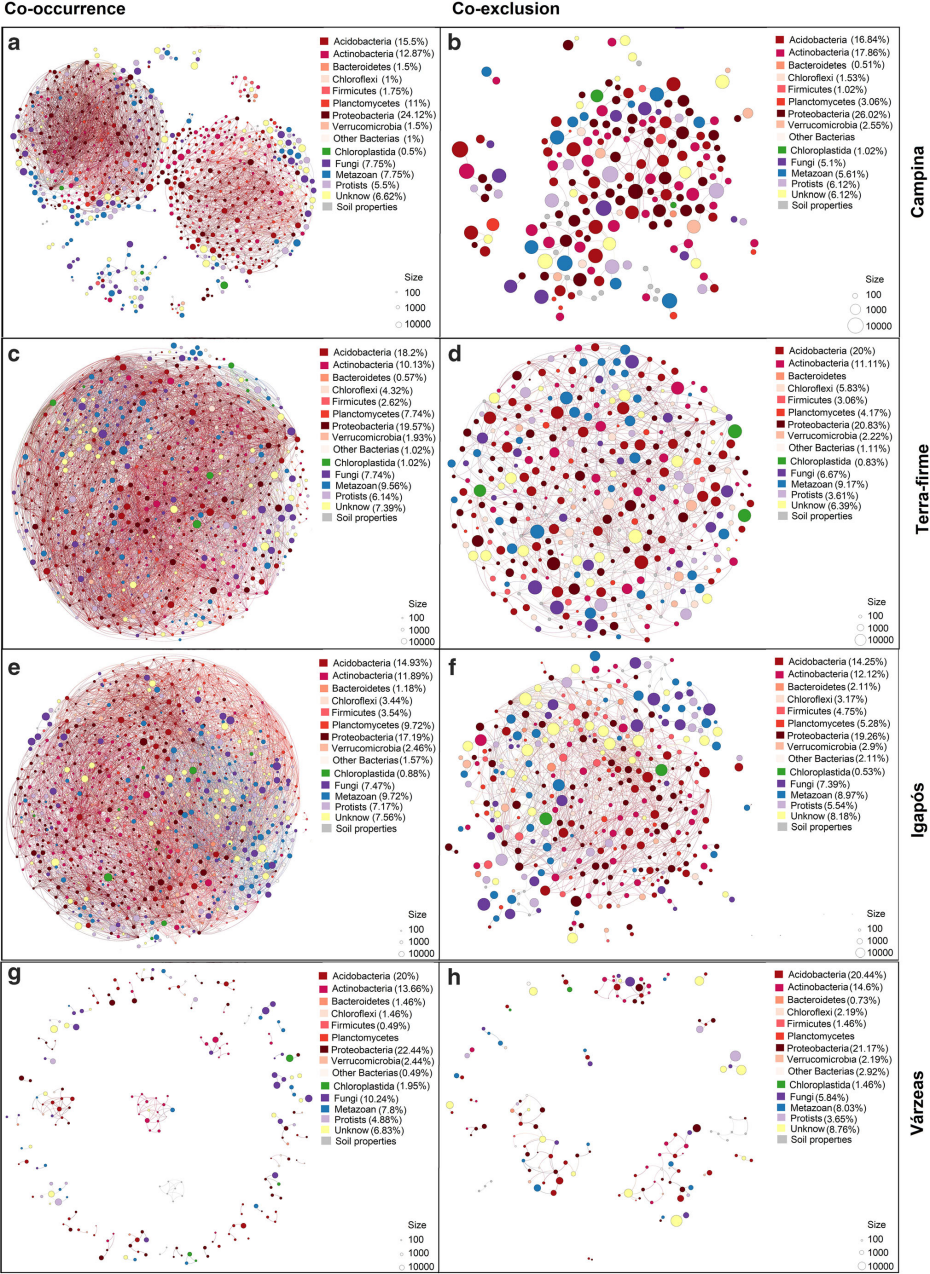
Soil community networks by habitat type. **a** Co-occurrence network and **b** co-exclusion network for campinas; **c** co-occurrence network and **d** co-exclusion network for terra-firme; **e** co-occurrence network and **f** co-exclusion network for igapó; **g**) co-occurrence network and **h**) co-exclusion network for várzea. Each OTU is represented by a node (circle) colored according to its taxonomic classification. The lines represent the edges connecting the OTUs. The size of each node represents the OTU abundance. Campinas are composed of two main network modules, while terra-firme and igapós are composed of one main network module. Várzeas have a co-occurrence network with very low complexity composed of fewer nodes and edges compared to the other habitats

Our third hypothesis (H_3_)—that habitat networks will show different network structures related to the presence of flood pulse as the main factor in explaining the network properties—was also partially supported. Campinas and terra-firme networks were more similar in structure and properties than igapós and várzeas (Fig. 4, Table 2). Várzeas showed by far the least complex co-occurrence network structure, but it was the habitat with the lowest number of replicates (12 samples, Fig. 4, Table 2). For the habitat networks within each locality, in general, there was a high modularity and transitivity, and a low average path length, diameter, and maximum components, with some regional variation (Table 2).

## Discussion

Due to the high diversity and turnover in Amazonia, we could expect a neutral community assembly due to dispersion limitation [72, 73] and a high redundancy of functional traits in the microbial community [74], which would result in less connected co-occurrence networks. However, here, we show that Amazonian microorganism communities form highly interconnected co-occurrence and co-exclusion networks when compared to those of other regions [12, 75]. For instance, global-scale samples of the Tara Ocean project, with 313 samples and more than 1 million OTUs, including viruses, showed a network composed of 29,912 OTUs and 127,000 edges with an average degree (number of edges per node) of 4.26 [76]. Our results, with a smaller sampling of 39 Amazonian plots (78 samples, 25% of the number of Tara Ocean samples) still resulted in a network of 72,577 edges involving 2350 OTUs, with an average degree of 30.88. In a study surveying soil bacteria in France, which included 30 soil samples for different land use categories (forest, grassland, crop system, and vineyards), researchers found a range from 16,430 edges involving 1083 nodes in forests to 2046 edges involving 1342 nodes in vineyards [12]. Our study comprised 24 samples for terra-firme, 24 for igapós, 18 for campinas, and 12 for várzeas, and ranged from 6223 edges involving 1047 nodes in terra-firme to 321 edges involving 273 nodes in várzeas. Although these studies use different methods to calculate the networks, our results demonstrate the relatively high complexity of Amazonian networks and highlight the importance of biotic and/or abiotic interactions in this region.

*H*_*1*_*: The Amazonian-wide network will be mostly composed by organisms associated with organic decomposition:* As expected in our H_1_, considering bacterial metabolism, there is a combination of bacteria that rely on respiration (e.g., Acetobacteraceae and Planctomycetaceae) and those that rely on fermentation (e.g., Fusobacteriales and Lactobacillales). These two groups complement each other in ecological functionality and indicate a high turnover of organic material underpinned by both metabolisms. Some groups present in the networks are active in degrading cellulose (e.g., Acidothermaceae and Polyangiaceae), which is in agreement with our litter samples that had a high amount of plant debris. There are also a few nitrogen-fixating groups, but they do not seem to be very common. Yet, one third of the bacteria recorded in our networks are not assigned to any functional group or preference, stressing the need for further investigation of Amazonian bacterial communities. Furthermore, the majority of fungi found here were saprotrophic, that is, decomposers of organic matter and, therefore, important agents in carbon cycling [77]. Unfortunately, archaea were heavily underrepresented in the results (just 131 OTUs) of our sequencing approach. We do not consider this a biologically meaningful result but rather an artifact of the amplification process, since archaea are commonly found in soil samples, where they contribute to, e.g., ammonia oxidation [78, 79]. We therefore decided to exclude archaea from our analyses and refrain from making any statements about this group of organisms.

Deforestation alters patterns of co-occurrence, impacting ecosystem functions [10]. The edges of Amazonian forest fragments have an increase of soil organic carbon probably due to high tree mortality [80], but potentially also due to an alteration of the microbial community [81], which may have an impact on the rate of organic decomposition and the ecosystem resilience capability. With the increasing anthropogenic pressures in Amazonia [82], it is crucial to understand how biological interactions are linked to decomposition in Amazonia and the potential effects of microbial community alteration in the world’s most diverse forest.

*H*_*2*_*: Environmental soil properties, especially pH and organic carbon (Ritter* et al. *2018), will be the most important factors to explain co-occurrence in all networks:* Our hypothesis H_2_ was partially supported. The organic carbon and base saturation indices were key nodes in explaining co-occurrence in the general network, whereas several other soil properties were key nodes for the co-exclusion, such as soil texture, exchangeable bases, aluminum, and pH. The composition of microorganisms in the soil is usually determined by physicochemical conditions such as phosphorous [83–85], pH [86, 87], and soil moisture [88]. Using the same data, Ritter et al. [27] showed that pH was the most important factor explaining the community turnover, and organic carbon the OTU richness [27]. This is the reason why we expected the pH to be important for the co-occurrence network, but instead we found it to be important just for co-exclusion.

Our results showed a predominance of bacteria that grow in low pH regimes, especially Acidobacteria [89], which are very abundant in soil. Amazonian soils are acidic, with pH varying from 3.65 to 5.14 [27], which explains the large presence of acidophilic organisms in both co-occurrence and co-exclusion networks. However, in less acidic soils, other groups could co-occur with acidophilic organisms, but the acidity tends to lead to increased competition. Phyla associated with low pH, such as Acidobacteria and Proteobacteria [85, 90], may be better competitors in more acidic soils. Furthermore, temperature appears to select for groups that are tolerant to high temperatures up to 40 °C (Fig. S8), which can occur in Amazonia all year. The soil properties were less important in the habitat-specific networks. As these soil properties are associated with the habitat types, and the same habitat presents similar soil physicochemical conditions [27], the network analysis of habitats may already be controlled by the variation in soil properties.

*H*_*3*_*: The habitat-specific networks will show different network structures, with the presence of flood pulse (the periodic inundation of floodplains along certain rivers) as the main factor explaining network properties:* Our hypotheses H_3_ was also partially supported. We expected that the high environmental stress in igapós and várzeas due to the seasonal flooding would act as an abiotic filter, keeping the same set of specialized organisms that co-occur for long periods of time, which would result in a dense and highly connected network. However, both habitat-specific networks were sparsely connected considering analyses within localities (Fig. S9–S12). Both habitats remain submerged during most of the year, up to 240 days [91]. In our data, the seasonally flooded forests—the várzeas and igapós—were more similar to each other in terms of community composition [36], which could be related to similar environmental filters linked to stress by flooding [92, 93], although the transitivity, that may indicate niche filtering, of the habitat networks was similar. Other factors, such as the random colonization due to smaller area size than in terra-firme, fragmented habitat distribution [94, 95], or the seasonally introduced species with the flood pulse that brings organism from river curse, could to some extent randomize the presence of OTUs. Várzeas differ from igapós by being a more fertile habitat, as their waters carry sediments from the Andes [92]. Therefore, a higher rate of colonization by microorganisms is to be expected in várzeas than in igapós, which are bathed by acidic, low-fertile waters [96, 97]. Várzeas may therefore have more microorganisms that could survive this particular stress condition, not just specialists. Indeed, várzeas had fewer specialist OTUs than igapós [36]. The difference between the sampling size of igapós (24 replicates) and that of várzeas (12 replicates), though, makes a more general comparison between these habitats difficult.

Communities in campinas (Fig. 4a) resulted in a smaller network (fewer nodes and fewer edges) than terra-firme (Fig. 4c) and igapós (Fig. 4e, Table 2), but larger than várzeas (Fig. 4g). However, terra-firme and igapós had more replicates (24 each) than campinas (18 replicates). Regarding network properties, campinas and terra-firme are similar (Table 2). However, the habitat network of campinas shows two distinct modules (Fig. 4a) that either represent two different metabolic pathways, as the dominant bacterial phyla are different in each module, or indicate that there is a geographical clustering [69]. The geographical clustering could be related to the natural fragmentation of campina habitats, which makes colonization more random than in more continuous habitats such as terra-firme [94, 95]. Yet, comparing the campinas networks within localities, the localities bathed by acidic, sediment-poor black waters (Jaú and Cuieras) had more modular networks (Fig. S9–S12, Table 2). That is also true for terra-firme, which had the low modularity (0.47 in co-occurrence network; Table 2), in other words a more connected network in Caxiuanã, a locality bathed by rich sediments from a white-water river (Fig. S9–S12). Stressful localities may have a more specialized co-occurrence community due to the environmental filters such as low pH, while less stressful localities may allow more organisms to co-occur.

### General Networks Composition

Overall, the key nodes of our networks are mainly affiliated to Proteobacteria (29 nodes, of which 17 belong to Alphaproteobacteria), Acidobacteria (19), Planctomycetes (12), and Actinobacteria (10, Table S3). These are common groups of bacteria in soils in general [52] and also in our samples [36, 37], being the most frequent groups in our networks. Alphaproteobacteria are a highly diverse clade of Gram-negative bacteria with several biological functions, including metabolizing C1 compounds [98], fixating nitrogen [99], endosymbiosis such as the widespread and important genus *Wolbachia* [100], and also intracellular pathogenicity [101]. Actinobacteria are important for the decomposition of organic matter from soils [102], making a range of nutrients available to other organisms, which probably explains the importance of this group in the co-occurrence network. Planctomycetes, a phylum of mostly aquatic free-living bacteria, but also found in soils [103], were also rich in our samples, with their distribution linked to soil properties such as soil organic matter, Ca2+, and pH [103]. Interestingly, even though Planctomycetes are associated with aquatic environments, they were not the richest in seasonally flooded forests (Fig. 4).

The co-occurrence network for all samples is densely connected, with low modularity and without differentiation explained by the habitat types, since most OTUs were classified as habitat generalists. If habitat type was the strongest variable to explain co-occurrence, the network should present modules, since groups belonging to a particular module should have similar environmental preferences [69, 104]. For instance, through analysis of deforestation along an Amazonian transect, it was possible to differentiate modules more associated with different degrees of deforestation [10]. The lack of modularity in the network for all samples may be due to the majority of OTUs (> 98% of nodes in all our networks) being classified as habitat generalists (present in three or four habitat types). The specialist OTUs (present in just one habitat) were usually local specialists, i.e., recorded in one or very few plots (Fig. 2), making the co-occurrence and co-exclusion patterns for specialist organisms hard to detect. Finally, even though networks for habitats are different from each other, the resulting pattern when including all samples may not show any apparent modularity, as patterns for each habitat are overlaid, and OTUs may present additional connections with OTUs from other habitats, smoothing or hiding patterns from individual habitats.

The high number of local specialist OTUs is in agreement with general microbial patterns elsewhere [105, 106] and also with distribution patterns of Amazonian tree species [95]. It is also in agreement with the competitive exclusion theory, which postulates that species with low competitive skills should be excluded from the community by highly competitive species [107]. Yet, these “poor” competitors remain in the communities as rare species [108]. In a global-scale study, rare species were found to have the highest positive association with each other, whereas common species had more negative associations [11]. However, that study relied on the number of observations of macro-organism species (plants and animals) and defined rare species in terms of low abundance. Abundance is difficult to quantify in metabarcoding studies due to PCR biases, such as the false negatives [109]. These biases will affect the detection of OTUs with low abundance beyond the intrinsic low detection probability and the stochastic distribution in their habitats due to their small abundance [110]. Yet, we had a relatively highly populated co-exclusion networks, which may be related to a high competition in soil communities, and further supports the exclusion theory in Amazonian microbial soil communities.

As microbial metabolic functions can differ or overlap, indicating metabolic complementarity or redundancy, they can explain either co-occurrence due to mutualism or co-exclusion due to competition [69]. For instance, several bacterial phyla are syntrophic, with complementary ecosystem functionality, such as microbial interactions for anaerobic methane oxidation, thermodynamic degradation, and nutritional exchange [111]. These associations potentially reinforce co-occurrence patterns among these groups and can explain the bacterial dominance in the co-occurrence networks. On the other hand, bacteria are also dominant in the co-exclusion networks, probably due to competition between other bacteria and also with fungi and protists. Examples of antagonist relationships between bacteria and fungi are well established [112, 113]. Most protists recovered in the networks feed on bacteria and some on fungi (or both), with few eukaryotic parasites recorded. Although this pattern is very different from what was found in other Neotropical soils [43], our results can be explained due to the prevalence of bacteria in our networks.

## Conclusions

We have shown that Amazonian soil communities have a densely connected network when compared with temperate forests or oceans. Bacteria dominate both the co-occurrence and co-exclusion networks. As some bacteria interact in ways that affect their growth and metabolism, these cooperative metabolic interactions can lead to increased growth of interacting bacteria and ultimately to positive co-occurrence patterns. On the other hand, competition for the same resources may lead to an inverse pattern and explain the dominance of bacteria in co-exclusion networks. Co-occurrence patterns may also reflect the response of different species to a common environmental factor rather than their direct interactions. All habitat networks in Amazonia were similar in structure and properties. Although co-occurrence networks cannot map out direct interactions in complex microbial communities, microbial network studies provide a way forward to understand potential intertaxon relationships in microbial communities.
